# A Qualitative Study of Stakeholders’ Perspectives of Implementing Interprofessional Shared Decision-Making Education in Palliative Care

**DOI:** 10.7759/cureus.44039

**Published:** 2023-08-24

**Authors:** Lama Sultan, Nynke de Jong, Basim S Alsaywid, Jascha de Nooijer

**Affiliations:** 1 Department of Clinical Nutrition, Ministry of National Guard Health Affairs, King Abdulaziz Medical City, Jeddah, SAU; 2 Department of Medicine, School of Health Professions Education, Faculty of Health, Medicine and Life Sciences, Maastricht University, Maastricht, NLD; 3 Department of Health Services Research, School of Health Professions Education, Faculty of Health, Medicine and Life Sciences, Maastricht University, Maastricht, NLD; 4 Directorate of Education and Research Skills, Saudi National Institute of Health, Riyadh, SAU; 5 Department of Urology, Pediatric Urology Section, King Faisal Specialist Hospital and Research Centre, Riyadh, SAU; 6 Department of Health Promotion, School of Health Professions Education, Faculty of Health, Medicine and Life Sciences, Maastricht University, Maastricht, NLD

**Keywords:** palliative care, shared decision-making, interprofessional education and collaboration, interprofessional shared decision-making, health professions education

## Abstract

Introduction

Shared decision-making (SDM) in palliative care is a highly complex process that requires an interdisciplinary team. Interprofessional team members need education on how to facilitate discussion of patient/family wishes at the end of life in hospital settings. So far, interprofessional shared decision-making (IP-SDM) education frameworks have been used to a limited extent in the area of education on palliative care. The aim of this study was to explore policymakers’, health professionals’, faculty members’, and students’ perspectives on implementing an IP-SDM educational framework in palliative care to identify aspects that should be prioritized to further develop interprofessional education for SDM in palliative care.

Methods

We used the qualitative method to capture the micro, meso, and macro factors using Oandasan and Reeves’ model for the implementation of IP-SDM education regarding palliative care. Data collection tools included in-depth, face-to-face interviews with individual policymakers and focus group interviews with health professionals, faculty members, and undergraduate health professionals. The interview guide explores the teaching of SDM in palliative care, factors that could facilitate or hinder the implementation of IP-SDM education for health professions students in palliative care, and interventions to facilitate the implementation of this approach. This study was conducted at the Oncology and Palliative Care Department at King Abdulaziz Medical City in the Ministry of National Guard Health Affairs and at King Saud bin Abdulaziz University for Health Sciences in Jeddah, Saudi Arabia.

Results

The results indicated a high demand for IP-SDM in palliative care. The findings revealed factors that can facilitate or hinder the implementation of IP-SDM education in palliative care for undergraduate health professions students that is going to the local community*. *Factors include culture, religion, gender, power issues, team hierarchy, and respect among team members. Also, our findings have revealed potential solutions to the hindering factors.

Conclusions

IP-SDM education in palliative care is a highly relevant topic for improving patient outcomes. However, it might be a complex process to implement, especially given the challenges of palliative care settings. We recommend starting such a course in the early clinical phases of undergraduate health professional education.

## Introduction

Given the aging population and growing numbers of life-threatening illness cases such as cancer, the demand for high-quality palliative care services is on the rise. The World Health Organization (WHO) has positioned palliative care as a public health issue [1]. The WHO defines palliative care as “an approach that improves the quality of life of patients and their families facing the problems associated with life-threatening illness, through the prevention and relief of suffering by means of early identification and impeccable assessment and treatment of pain and other problems, physical, psychosocial, and spiritual” [2]. The aim of palliative care is to enable patients to live as actively as possible and to offer support to the family during the patient’s illness [2].

One of the barriers to the effective delivery of palliative care includes insufficient training in end-of-life care communication skills [3] and in shared decision-making (SDM) [4]. SDM is a process of integrating both the best available evidence and patients’ values and preferences when the patient/family and health care team discuss decisions regarding the patient’s care [5]. Given the complexity of palliative care, SDM requires an interdisciplinary team [6]. The team must collaborate and communicate effectively to provide high-quality care for patients and their families and to involve them in the SDM process [7]. Palliative care is provided by a team of different health professionals, including doctors, nurses, social workers, spiritual care practitioners, psychologists, pharmacists, and allied health practitioners, who work collaboratively with the patient and/or family to improve the patient’s quality of life [8]. The members of such interprofessional teams need education on how to facilitate discussions with patients and their families at the end of life and to provide ongoing support and care [9]. However, there is a lack of educational programs based on theoretical insights into interprofessional education (IPE) and SDM [4,10]; therefore, the effectiveness of such education remains unknown [11,12].

Reviews have highlighted the need for education that focuses on IPE in palliative care in which students, patients, and their families are members of the IPE teams [13,14]. Also, incorporating skills training for SDM in teaching settings is needed to prepare students for clinical practice [15]. This need remains, even though the processes for implementing IPE have been outlined in the literature (e.g., in the National Academy Press [16]). The obstacles to implementing IPE include logistic, curricular, and cultural factors and depend on the leadership and faculty. Although palliative care is interdisciplinary by nature, implementation of IPE in palliative care faces various barriers, including logistic issues, lack of faculty experience in IPE, absence of evaluation, and limited evidenced-based education for promoting interprofessional teams and interdisciplinary care [17].

Currently, palliative care is considered a key national and regional strategic priority in Saudi Arabia, as the cancer burden is expected to grow in the next 10 years due to unhealthy lifestyles and the aging population [18]. Palliative care was one of the major elements the Ministry of Health discussed in its plans for Saudi Vision 2030 [19]. To date, palliative care education has received little attention in Saudi Arabia. Also, the number of interventional studies on IPE and IPE programs that have been implemented is limited [20]. However, students and faculty have demonstrated positive attitudes regarding the importance of teamwork and collaboration through IPE [21]. No previous research has addressed interprofessional shared decision-making (IP-SDM) and palliative care education in Saudi Arabia. Therefore, the aim of this study is to explore the policymakers’, health professionals’, faculty members’, and students’ perspectives on implementing an IP-SDM educational framework in palliative care at King Abdulaziz Medical City in Jeddah, Saudi Arabia, to identify aspects that should be prioritized when developing IPE for SDM in palliative care.

## Materials and methods

Study area/setting

This study was conducted at the Oncology and Palliative Care Department at King Abdulaziz Medical City in the Ministry of National Guard Health Affairs in Jeddah, Saudi Arabia, which is a 751-bed hospital serving Ministry of National Guard employees and their families. The study was also conducted at King Saud bin Abdulaziz University for Health Sciences in Jeddah, Saudi Arabia.

Study design

The study design encompasses a qualitative method that consists of two data-collection tools: in-depth, face-to-face individual interviews and focus group interviews. To guide IPE planning, we adapted the interprofessional education for collaborative patient-centered practice (IECPCP) model for this study [22]. The model is based on a systematic review conducted for Health Canada IECPCP projects and provides factors for the success of IPE [22]. The model has two interdependent components: (1) interprofessional education and (2) collaborative patient-centered care.

It was designed to bridge the gap and establish linkages between educational initiatives that enhance learner outcomes and collaborative practices that enhance patient outcomes. The processes of collaboration have micro, meso, and macro levels [22]. Micro-level factors (teaching factors) include interprofessional planning for IPE, faculty development, and student engagement; meso-level factors (institutional factors) include leadership support, organizational structure, and administrative challenges; and macro-level factors (systematic factors) include partnership with government and practice communities and evaluation and quality improvement (Table 1). We used this model to assess factors that facilitate or hinder the implementation of IPE and to identify who needs to be involved and what needs to be addressed in the implementation of an IP-SDM education initiative in palliative care by addressing the three levels.

**Table 1:** Key factors addressed at the micro, meso, and macro levels. IP-SDM: interprofessional shared decision-making

Micro level (teaching factors):
Educational theory for IP-SDM in palliative care; interprofessional planning for IP-SDM education in palliative care; student engagement; faculty development.
Meso level (institutional factors):
Leadership support; organizational structure; IP-SDM coordinator; dedicated resources; strategic plan.
Macro level (systematic factors):
Partnership with government and practice communities; evaluation and quality improvement.

Study subjects 

Policymakers included the head of the Palliative Care Department and deans of the College of Medicine, the College of Nursing, and the College of Applied Medical Sciences. We included health professionals and faculty members from medicine, nursing, clinical nutrition, respiratory therapy, and occupational therapy. We also included undergraduate students of health professions in the following colleges: the College of Medicine, the College of Nursing, and the College of Applied Medical Sciences (respiratory therapy, occupational therapy, and clinical nutrition). Students of both genders were included. Participating students had to be current students enrolled during the 2021-2022 academic years who were interns or seniors, which means years 5 and 6 for the College of Medicine and years 3 and 4 for the College of Nursing and Applied Medical Sciences. All participants who fulfilled the above criteria were invited personally via email to participate in this research. The email contained the aim of the study. All participants who showed interest and availability to participate in either the individual interviews or the focus group interviews read and signed a consent form. Subsequently, an email confirmation was sent, explaining the date, time, and location of the interviews.

Sample size and sampling technique

We used the nonprobability purposive sampling technique to secure information-rich participants for policymakers, faculty members, and health professionals, whereas we used the nonprobability convenience sampling technique for senior health professions students. Participants were recruited from the following categories: (i) policymakers (n=5); (ii) faculty members and health professions staff from interprofessional teams who have an impact on health care decisions (n=15); and (iii) senior health professions students (n=25). 

Data-collection methods

The IECPCP model proposed by Oandasan and Reeves, which describes the various factors and elements underlying and influencing interprofessional practice, was used to guide the open-ended interview questions [22]. The interview guide captures the micro, meso, and macro levels of stakeholders’ perspectives on IP-SDM education in palliative care (Tables 2, 3, 4). 

**Table 2:** Interview guide for policymakers. IP-SDM: interprofessional shared decision-making

Introductory questions:
1-Do you have experience with SDM in healthcare? 2-What does IP-SDM in palliative care mean to you? 3-Are you aware of any program or course that addresses IP-SDM in palliative care nationally?
Micro-level questions:
4-What competencies (knowledge, skills and attitudes) must be taught in IP-SDM in palliative care? 5-When should IP-SDM be introduced into health professions education? Why? 6-How should we educate our health professions students in IP-SDM? 7-What possibilities do you see for health professions students to learn with, from and about each other in palliative care? 8-What hindrances do you see for health professions students to learn from each other in palliative care? 9-What aspects are important with respect to assessment of SDM in health professions education? 10-How can we prepare faculty to facilitate IP-SDM in palliative care? 11-What can policymakers do from their positions within the university to facilitate IP-SDM for learners and faculty?
Meso-level questions:
12- What staffing and support resources are needed for the implementation of IP-SDM in palliative care in your institute? 13-What changes must be made in the curriculum to implement SDM in health professions education? 14-How can colleges coordinate their IP-SDM education courses? 15-What elements of the culture within your institute support IP-SDM education implementation? 16-What external or internal factors influence the development of IP-SDM education in palliative care?
Macro-level questions:
17-What barriers to implementing IP-SDM teaching in palliative care do you anticipate, and how can you overcome them? 18-What is necessary to ensure the sustainability of IP-SDM teaching in palliative care? 19-How can the impact of IP-SDM education be measured? 20-What accreditation standards are needed relating to IP-SDM education for health professions programs? 21-Can you recommend any experts in the field of IP-SDM education that we need to involve in the study?
Closing questions:
22-Is there any additional information you’d like to share regarding IP-SDM education in palliative care in general or within your institute? 23-Is there anything else that you consider important regarding the design and implementation of IP-SDM within your institute that has not yet been addressed? 24-Do you have any other remarks or comments?

**Table 3:** Focus group interview guide for health professionals and faculty members. IP-SDM: interprofessional shared decision-making

Introductory questions:
1-Do you have experience with SDM in health care? 2-What does IP-SDM in palliative care mean to you? 3- Are you aware of any program or course that addresses IP-SDM in palliative care nationally?
Micro-level questions:
4-What competencies (knowledge, skills and attitudes) must be taught in IP-SDM in palliative care? 5-Can you give examples of intended learning outcomes for IP-SDM education programs/courses in palliative care? 6-What teaching and learning strategies can be used to teach IP-SDM in palliative care? 7-When should IP-SDM be introduced into health professions education? Why? 8-How can we engage students in IP-SDM education preparation and implementation? 9-What possibilities do you see for health professions students to learn with, from and about each other in palliative care? 10-What hindrances do you see for health professions students to learn from each other in palliative care? 11-What aspects are important with respect to assessment of SDM in health professions education? 12-What type of faculty development is needed? How could the faculty be prepared to facilitate IP-SDM in palliative care? 13-What can policymakers do from their positions within the university to facilitate IP-SDM for students and faculty?
Meso-level questions:
14-How can you incorporate SDM in health professions education in palliative care in your program/university? 15-What staffing and support resources are needed for the implementation of IP-SDM in palliative care in your institute? 16-How can colleges coordinate their IP-SDM education courses? 17-What are the challenges of IPE in SDM in palliative care in your curriculum/university/institute? 18-What elements of the culture within your institute support IP-SDM education implementation? 19-What external or internal factors influence the development of IP-SDM education in palliative care?
Macro-level questions:
20-What barriers to implementing IP-SDM teaching in palliative care do you anticipate, and how can you overcome them? 21-What is necessary to ensure the sustainability of IP-SDM teaching in palliative care? 22-How can the impact of IP-SDM education be measured?
Closing questions:
23-Is there any additional information you’d like to share regarding IP-SDM education in palliative care in general or within your institute? 24-Is there anything else that you consider important regarding the design and implementation of IP-SDM within your institute that has not yet been addressed? 25-Do you have any other remarks or comments?

**Table 4:** Focus group interview guide for health professions students. IP-SDM: interprofessional shared decision-making

Introductory questions
1-What do you know about SDM in health care? 2-What do you know about palliative care? 3-What do you know about IPE? 4-What does IP-SDM in palliative care mean to you? 5-Are you aware of any program or course that addresses IP-SDM in palliative care nationally?
Micro-level questions:
6-Have you participated in any IPE session/course/program? If so, what was your experience like? 7-What competencies (knowledge, skills and attitudes) must be taught in IP-SDM in palliative care? 8-How can you learn from different disciplines in palliative care? 9-What possibilities do you see as health professions students to learn with, from and about each other in palliative care? 10-What hindrances do you see as health professions students to learn from each other in palliative care? 11-How do you want to learn in IP-SDM in palliative care? 12-When should IP-SDM be introduced into health professions educational programs? Why? 13- How can you as a student be engaged in IP-SDM education design construction, evaluation, and development? 14-What assessment tools do you expect for SDM in health professions education? 15-What can policymakers do from their positions within the university to facilitate IP-SDM for students?
Meso-level questions:
16-How can SDM in palliative care be incorporated in health professions education in the university? 17-How can colleges coordinate their IP-SDM education courses? 18-What external or internal factors influence the development of IP-SDM education in palliative care?
Macro-level questions
19-What barriers to IP-SDM teaching in palliative care do you anticipate, and how can you overcome them? 20-What is necessary to ensure the sustainability of IP-SDM teaching in palliative care?
Closing questions:
21-Is there any additional information you’d like to share regarding IP-SDM education in palliative care in general or within your institute? 22-Is there anything else that you consider important regarding the design and implementation within your institute that has not yet been addressed? 23-Do you have any other remarks or comments?

Interviews and focus groups were conducted in October and November 2021. Interviews with policymakers lasted for 45 to 60 minutes. Focus groups were divided cohesively into health professionals and faculty members or students according to discipline. Each focus group interview consisted of four to 10 members. All interviews were conducted in English and were audio recorded. The setting and the time frame for each focus group interview were similar (a round table in a meeting room and 90 minutes per group). The main researcher (LS) conducted all the interviews.

Data analysis

The main researchers (LS) transcribed all audio-recorded interviews verbatim. Transcripts were uploaded into Quirkos 2020 (a qualitative analysis software package; Quirkos, Edinburgh, UK) to manage and organize the data. The transcripts were coded after the research team (LS, JN, NJ, and BA) read them several times. A provisional thematic framework was developed by using Quirkos. The research team analyzed and discussed all data. 

## Results

In total, five individual interviews with five policymakers were conducted, and six focus group interviews were conducted with 40 participants divided into the following groups: medicine faculty and staff (n=4), nursing faculty and staff (n=6), applied medical sciences faculty and staff (n=5), medical students (n=10), nursing students (n=8), and applied medical sciences students (n=7). The majority of participants were Saudis. Regarding participants’ gender, 31 were females and 14 were males. Regarding their specialty, 16 participants were from medicine, 15 were from nursing, and 14 were from applied medical sciences (Table 5). 

**Table 5:** Characteristics of participants.

Characteristics		All participants (n=45)
Age by years (min-may) mean ± SD		11.4±31.5 (57-21)
Gender	Male	14
	Female	31
Nationality	Saudi	43
	Non-Saudi	2
Specialty	Medicine	16
	Nursing	15
	Applied Medical Sciences	14

This paper focuses on stakeholders’ perspectives of implementing IP-SDM education in palliative care by applying the IECPCP model. Five major themes emerged as factors that facilitate or hinder the implementation of IP-SDM education in palliative care for undergraduate health professions education. We have provided potential solutions for overcoming these hindering factors. For insight into the nature of the data contained in these themes, extracts of conversations are presented. These extracts were chosen to represent and illustrate each of the themes (Table 6). The facilitating factors included the relevance of IP-SDM for palliative care and awareness of its importance (systematic factor). The hindering factors included considerations of culture, religion, and gender (systematic factor); issues related to the content and implementation (institutional factor); issues related to the staff (teaching factor); and considerations of team members' respect, hierarchy, and power (teaching factor).

Facilitating factors of implementing IP-SDM education in palliative care

With respect to the relevance of implementing IP-SDM education in palliative care for health professions students, many interviewed professionals reported that the demand for and number of patients needing palliative care are increasing (also see Appendices). They were aware that the Saudi Ministry of Health considers palliative care as one of the six dimensions and a major element of its plans for Saudi Vision 2030. 

Participants agreed on the importance of the IP-SDM and its application in the field of palliative care. One of the policymakers mentioned that the application of this project aligned with the university research criteria. Despite the relevance of the topic, one of the policymakers indicated that implementation would not be easy. Our findings indicated factors that can facilitate the implementation of IP-SDM education for health professions students in palliative care. Such factors include the availability of the Oncology and Palliative Care Center, simulation center, and medical education experts.

Hindering factors of implementing IP-SDM education in palliative care

Culture, religion, and gender are factors that participants indicated must be considered when implementing IP-SDM education. Because these factors influence patient/family decision-making, students emphasized the importance of addressing these factors in IP-SDM education for palliative care. One of the policymakers referred to culture as an obstacle or boundary that must be respected. Health professions students need to know how to assess patients according to their culture and religion and seek to understand their needs. They also must consider gender role differences and belief systems in palliative care settings.

Participants felt that IP-SDM in palliative care had value and needed to be integrated into health professions curricula, preferably in longer learning trajectories to achieve better clinical learning outcomes. However, there were issues related to the content and implementation, such as the difference in place of the palliative care course within various curricula (medicine, nursing, and allied health), the availability of the course, and whether it is mandatory in one curriculum and elective in another.

The professional system acknowledges the need for SDM in palliative care, and this is also supported by the Saudi Vision 2030, but implementing IP-SDM in palliative care education requires additional efforts from the staff. There were concerns related to staff not being prepared for IPE or IP-SDM, staff not accepting a role as a facilitator instead of an expert teacher, and staff themselves not having training, which makes it difficult for them to switch to IPE. Team member respect, team hierarchy, and power issues were addressed by almost all participant groups as the main factors to be considered when implementing the IP-SDM teaching method in palliative care. The participants discussed this issue in both education and real practice.

Potential solutions for mitigating the hindering factors

The hindering factors of implementing the IP-SDM in palliative care for health professions education included consideration of culture, religion, and gender; issues related to content and implementation; issues related to staff; and considerations of team member respect, hierarchy, and power. This section addresses the potential solutions for the abovementioned hindering factors (also see Appendices). Faculty members had solutions for overcoming barriers related to culture, religion, and gender, by considering those factors when developing the intended learning outcomes for IP-SDM education in palliative care. It was recommended that students attend multidisciplinary meetings to better understand other perspectives and to learn about culture, religion, and gender factors that influence IP-SDM in palliative care settings. 

Policymakers had suggestions for how to overcome difficulties related to content and implementation regarding the place and timing of IP-SDM in palliative care for health professions education. Policymakers suggested adding the palliative care topic to all health professions curricula and making IPE an educational strategy in the university. Policymakers and faculty members also had ideas for how to overcome issues surrounding the implementation of teaching methods for IP-SDM education. For instance, they suggested including solving complex tasks that require teamwork from different disciplines in bedside teaching, learning by doing, community-based learning, and simulation.

Participants expressed their preferences for the faculty development program and to learn from international sources for developing the faculty development program and IP-SDM courses to overcome issues related to staff. Furthermore, policymakers’ support for such initiatives is considered critical in the initial implementation of these types of courses.

To create a safe learning environment and overcome power issues, policymakers suggested introducing the concept of team hierarchy and teamwork early in undergraduate education, attending multidisciplinary meetings and grand rounds in palliative care, building an IPE environment, utilizing the simulation center to facilitate such education, and learning by doing. Both faculty members and students indicated the importance of team respect in SDM and emphasized integrating this concept in teaching in undergraduate health professions education. To overcome issues related to team members' respect, team hierarchy, and power dynamics, the majority of participants suggested addressing the roles of each team member and developing effective communication skills to avoid conflict in palliative care settings.

## Discussion

The aim of this study was to explore stakeholders’ perspectives about the factors that facilitate or hinder implementing IP-SDM education in palliative care. We used the IECPCP model as the basis for our study. We addressed systematic, institutional, and teaching factors. We focused on five main themes that emerged as factors that facilitate or hinder the implementation of IP-SDM education in palliative care: relevance of the IP-SDM topic (systematic factor); considerations of culture, religion, and gender (systematic factor); issues related to content and implementation (institutional factor); issues related to staff (teaching factor); and considerations of team members respect, hierarchy, and power (teaching factor). Strategies for overcoming the hindering factors have been addressed in the literature [17]. It is suggested that these learned lessons be translated to the context of interprofessional collaboration and IPE in Saudi Arabia and that results be communicated to the wider community [23].

Regarding the systematic factors, participants expressed the need for IP-SDM education in palliative care given the increased number of patients and Saudi Vision 2030, but participants were concerned about the implementation of IP-SDM in practice. Similar attitudes were found in another study on implementing IPE in Saudi Arabia, which showed that faculty supported IPE and appreciated its long-term benefits but were aware of the barriers to its implementation and emphasized creating a supportive environment with the involvement of students, faculty members, and top University administrators [24]. Another study on end-of-life nursing education suggested incorporating diverse beliefs, religions, and cultures in the implementation of palliative care education [25], especially because dealing with the end of life is a sensitive situation that is overwhelming for patients, family members, and caregivers [26]. Religion can be a hindering or facilitating factor due to specific aspects of Islam that have contrasting elements of taking care of others and respecting end-of-life. Also, gender is a hindering or facilitating factor as women tend to enroll in end-of-life more than men [27]. Culture remains a notable barrier to collaboration in education and practice. The Saudi Arabian community is framed by tribal bounds and greater religious constraints when compared to Western settings [28]. To overcome barriers related to culture, it is recommended by policymakers to integrate problem-based curricula that emphasize IPE [16] so that the focus of education is on solving problems in a particular context. Changing the culture of an organization requires support from executive leadership [16, 29]. In this study, policymakers agreed on the importance of the topic, especially given its alignment with university criteria and Saudi Vision 2030, which highlights the importance of engaging in IPE and collaboration to provide optimal care in health care facilities.

Regarding institutional factors, because IPE and palliative care are not in all undergraduate health professions curricula, it is suggested by policymakers to find the right timing for IPE and the right match of learners among professions as well as in the physical space. Our findings demonstrated a great need to incorporate IP-SDM education in palliative care as early as possible in the clinical phases of undergraduate health professions education, when students have acquired basic knowledge about their professions and developed professional identities. Furthermore, the description of our organizational structure presents opportunities to standardize IPE efforts across schools and health systems. The participants in our study emphasized teaching interprofessional collaborative practice skills through learning by doing, simulation, and briefing and debriefing. The suggestion to use simulation as an educational strategy is supported by other studies [30-32]. Therefore, simulation is an appropriate strategy for improving graduates’ ability to manage patients collaboratively and interact with each other in real practice. Furthermore, some undergraduate students in our institute participated in health awareness campaigns from different health professions disciplines, such as cancer day, to produce an educational program for the community. So, using the same strategy for implementing IP-SDM in palliative care makes sense when the content relates to meaningful clinical and community experiences. With regard to curriculum content, it is suggested by faculty members that educators weave the content into meaningful clinical and community experiences [16].

Regarding the teaching factors, not all faculty members are able to provide IPE or are trained to coach and assess different types of health professions students. Our findings suggested that faculty development in palliative care, interprofessional teaching skills, and interdisciplinary team collaboration competencies are desired. Participants addressed the need for faculty members to receive training on how to teach IP-SDM in palliative care so they can be prepared intellectually. Researchers have shown that faculty development has a positive effect on IPE preparedness and engagement [33-35]. For instance, El-Awaisi et al. proposed a 12-step guide to support the preparation of IPE in health professions education [36]. The first step focuses on stakeholder perceptions and faculty development, which are essential factors for the success of IPE initiatives. Faculty development has been addressed in literature [16], and it is suggested that seminars and workshops be conducted for clinical and educational faculty. Furthermore, a training program in palliative care that addresses barriers and facilitators when setting up palliative care services is needed [37, 38].

In our study, participants frequently cited power issues and team hierarchy as core elements in the implementation of IP-SDM education in palliative care. According to Interprofessional Education Collaborative (IPEC) Core Competencies for Interprofessional Collaborative Practice [39], values/ethics for interprofessional practice, roles and responsibilities, interprofessional communication, and teams and teamwork are the four competencies of Interprofessional Collaborative Practice. To reduce power issues, staff should start with the same foundational knowledge, so that knowledge discrepancies are not the reason for issues of power and hierarchy [40]. The same content knowledge was found to help overcome stigmatization and power issues. Teaching teamwork and conflict resolution and addressing the role of each health care team member are necessary to make people aware of each other’s responsibilities and to provide high-quality health care. Researchers suggested developing IPE by applying landscapes of practice principles [41] and focusing on engagement, imagination, and alignment to enable students’ identity formation.

This study has a number of limitations. First, it was based on one institute in a particular context, which limits the generalization of the findings to other contexts. However, because our study represents different colleges with different cultures, we believe we covered the full scope of beliefs related to the implementation of IP-SDM education in palliative care. To overcome the barriers related to the local context, we formulated solutions that are also applicable in other contexts. Second, the use of purposive sampling and the particular characteristics of the participants make our study highly prone to selection bias. We included all the relevant stakeholders - policymakers, health professionals, faculty members, and undergraduate health professions students - from the institute where we are going to implement the IP-SDM in palliative care for health professions education.

The novelty of this study is that it focuses on IP-SDM in the palliative care setting, whereas many IPE initiatives focus on chronic diseases. Palliative care is a sensitive topic, and dealing with the end-of-life decisions of patients and/or their caregivers creates ethical challenges for health care. In addition, this study is relevant to the Saudi Arabian community, which has its own social, religious, and cultural challenges that no paper has addressed before. The tribal diversity in Saudi Arabia also adds an extra dimension to the field of IPE and SDM in palliative care.

## Conclusions

Our findings indicated that IP-SDM education in palliative care is relevant and timely but complex to implement, especially given the challenges inherent to palliative care settings. Our findings revealed educational, institutional, and systematic factors that should be addressed when implementing IP-SDM education in palliative care for undergraduate health professions students and applied to the local community. The impact of tribal diversity in Saudi Arabia adds an extra dimension to the field of IPE and SDM. Based on the findings of our study, we recommend starting such an educational course as early as possible in the clinical phases of undergraduate health professions education. This study might also serve as a useful needs assessment for health professions educators planning to implement curricula around IP-SDM in palliative care for undergraduate health professions students.

## Appendices

**Table 6:** Extracts from participants' quotes by each theme. IP-SDM: interprofessional shared decision-making; IPE: interprofessional education;

Facilitating factors of implementing IP-SDM education in palliative care
“We started to see increasing numbers of patients of this category in the last decade, and I think this specialty is increasing with the number of patients. Therefore, the students will encounter these kinds of patients and fields more frequently rather than or compared to the previous health care practitioners.” (Policymaker 3) “The demand is increasing, the number of patients is increasing, and the vision does support the needs.” (Policymaker 3) “My work as a geriatrician for the last 16 years and in palliative care exposed us to a point that you need to share decisions regarding patient care with the other team, with other disciplines and with the patient and their families.” (Policymaker 2) “..we have to keep in mind that the new module that the Ministry of Health is going through, which includes one of the six pathways, is palliative care. So, this is, I think, a big element that are influences us to push toward doing that.” (Medicine faculty 1) “If we will talk about our university, the domains are known, which is knowledge, research and community service, and I see this under the three of them. As a knowledge, as a good area of research, and it’s a community service. This project fitted completely the criteria.” (Policymaker 5) “We should now switch gears into specific patient management, not just disease- focused. So patient-oriented evidence and intervention that matters.” (Policymaker 2) “I totally agree that the shared decision-making is very important. But how to implement this? We have to be careful about the implementation.” (Applied medical sciences faculty 3) “Internal, we have Oncology Center, where you have a lot of cases and workloads on oncology and palliative care increasing. So that’s a factor I think it would help to push.” (Policymaker 2) “..and so what I said for it to be done in the undergraduate fashion….. Because there is an oncology center, simulation center and experts in medical education.” (Policymaker 4)
Hindering factors of implementing IP-SDM education in palliative care
“Changing the culture is very difficult. When you are continuously training the faculty, you can change the culture. By default it will change.” (Policymaker 1) “... Gender is also another example.” (Medicine faculty 1) “Caring of the caregiver, because they are dealing with such an advance and such a terrific time with their loved one. Also, … understanding the culture and belief and religion of our patients— this might be added.” (Nursing faculty 2) “..which you mentioned about professional level might be good to consider as well on a personal level, considering religion ..” (Medicine student 1). “Now the idea of having it professionally shared decision-making is becoming more fashionable here.” (Policymaker 2) “One of the things is the palliative care is not in all curricula. May be in nursing, medicine, but not in physiotherapy curriculum. This is one of the issues.” (Policymaker 1) “Different experience in knowledge because we are as nursing students, we learn the palliative care in some aspect, but there are medicine students, for example, they are involved really in the course, but not superficial. Yeah. so, we can see gaps in knowledge. And it’s an elective course in nursing.” (Nursing student 2) “Palliative course should be as a mandatory course in nursing, not elective, because in real practice there are many palliative patients. If some nursing students didn’t take the course, how will they deal with the palliative patients?” (Nursing student 2) “.. We take palliative care course, but not IPE.” (Applied medical sciences student 3) ''…The other issue is, as I said, preparation of the faculty. Not all the faculty are prepared to do interprofessional education. They need to have enough knowledge and skills and competency in teaching the interprofessional to palliative care shared decision-making.” (Policymaker 2) “The barrier is people accepting the role to be a facilitator or a participant and the different personality related to specialty or related person.” (Policymaker 3) “ First we have to change the people’s mind. Many people find is very difficult to change, like if I want to teach interprofessional, I have to have the people from the clinical nutrition department being very willing to teach the nursing department and the medicine and all together.” (Policymaker 1) “Interprofessional is really a mind switch. I’m a nurse, I’m teaching only nurses. No, that is not true. You are teaching. You can teach everyone skills.” (Policymaker 1) “You cannot teach interprofessional decision-making without interprofessional education.” (Policymaker 4) “ You need to prepare the people intellectually on how to teach interprofessional.” (Policymaker 4) “I think to be honest with you that the concept of the interprofessional is a new concept. We’ve graduated from college and such a term didn’t hear about it. We really need to introduce this term in faculty senior people and to recruit them and to get them engaged in such a new concept.” (Medicine faculty 1) “..if we built our system based on interprofessional education, it would be much easier. Also, the problem of who’s going to be the boss?” (Policymaker 1) “The whole problem of undergrad that we graduate very competent physicians, very competent physiotherapists, very competent occupational therapists, etc., very competent clinical dietitians, but we don't teach them how to manage things together as a team, how to interact, what is the intersection, the real ability of other health care workers’ specialties and limitation.” (Policymaker 1) “…knowing how to be part of the team, knowing how to lead the team, knowing the dependencies between the team members and thinking about the patient at the end, which is sometimes a really tough formula to achieve.” (Policymaker 3) “I will concentrate in collaboration, especially for function of the team and resolve the conflicts. And I really think as a palliative doctor, these are the most things you are facing: learning how to function with the team and how to resolve the conflict.” (Medicine faculty 1) “.. we have a lack of exposure with other health care professionals. Our students graduate from the program, they are full loaded with knowledge, but they don’t know the roles and responsibilities of other health care professionals.” (Applied medical sciences faculty 3)
Potential solutions for mitigating the hindering factors
“I want to add also, different culture considerations.” (Applied medical sciences faculty 3) “Education wise, actually, I'm doing the monthly meetings in Arabic Culture Week for the newcomers, staff nurses about the palliative in Kingdom”. (Nursing faculty 2) “They will also need to know about the other perspectives, such as social aspect, culture aspect, gender, and religion. So, they will know a lot of things regarding seeing each other on a daily basis or even dealing with each other during the meeting.” (Nursing faculty 2) “I think the policymakers should ensure that the curriculum has a good part of focusing on managing patients, tailor management, quality of life, sharing part of the curriculum with other disciplines as for undergrad.” (Policymaker 2) “I think when people have the basic knowledge of their profession, like if they have a basic knowledge of nursing and then the basic knowledge of like clinical nutrition, the basic knowledge of medicine, then we have to introduce the interprofessional education, because if they do not know their own role in their profession, then they do not know how to work interprofessionally… So, I think it has to be introduced the time they introduce the profession knowledge itself. So, it doesn’t have to be in the late phase of each of any program; it has to be in the middle as early as possible.” (Policymaker 1) “.. having an agreement also with the hospitals for the internship students to get a mandatory, I would say, rotation or short rotation for palliative care patients. For example, one, one or two weeks, whatever the hospital is. So, maybe yes, if they get that, this will be a great opportunity for them to prepare them well in how to deal with these kinds of patients.” (Applied medical sciences faculty 3) “Completing each other is a good method if they feel they complete each other in their learning. They succeed very well. They learn very well, practicing everything. And like by clinical training, especially in palliative care, clinical training or bedside training is very useful. They learn from each other, and they learn different techniques. Also, they learn by doing, which is usually a very successful way of learning.” (Policymaker 1) “ I have seen students also are participating in complicated disease, cancer day, or cancer awareness. And then they have to work together to come up with a good education program for the community.” (Policymaker 2) “Utilizing simulation in doing these communication skills or building scenarios that would help the interprofessional decision-making process that would test the team, and they can do that in a simulation fashion and then utilizing briefing and debriefing after that.” (Policymaker 4) “…You have to train faculty, and we have to give them the chance to practice it gradually. First of all, you have to have the qualified staff—staff who are qualified to be or trained in palliative care and trained in interprofessional education.” (Policymaker 1) “Learning from international experience, attending some conferences, even if it's online, regarding the interprofessional palliative care patient management.” (Policymaker 2) “I would make sure that our faculty enhancement unit to work together to develop this course with the resources available at the college level...If you had asked me, I would say yes, I would love to have to see this course for our faculties, including myself, to learn about shared decision-making.” (Policymaker 3) “I think if we’re thinking about specifically palliative care. The grand round and the multidisciplinary round would be a good opportunity to come to a common understanding between the faculty….Having grand round, in the journal club, also looking at studies and measuring the achievement of our patients, for example, the length of stay for palliative care patients after the multidisciplinary team approach.” (Policymaker 2) “I will focus on collaboration in interprofessional teaching how to function with the team. Knowing the teams, knowing the function in the team and the limit of each one, and knowing and resolving their conflicts, and then knowing when to become assertive to become collaborative.”(Medicine faculty 1) “We have to break down those barriers and explain to people that everyone has a job, everybody has a role, and we’re always important to each other and, of course, to the patient. Everybody brings something to the table that's important. And I think some people come to the table without having that idea in their mind.” (Nursing faculty 2) “ Teach respect, cooperation, confidence to share your thoughts and decisions.” (Applied medical sciences faculty 3) “ The most important thing is how to deal with team teamwork, other teams, and how or what values and ethics we should incorporate in the program, like respecting patient values, what he needs, what he thinks of.” (Medicine student 1) “Let's say suboptimal communication between the professions. Um, we are talking about the understanding the importance of the role of each team member.” (Policymaker 5) “ Being in emergency seeing a lot of patients, I am talking about my experience as an emergency physician. We see a lot of patients, that could be all avoided if proper communication among health care professions is done in advance.” (Policymaker 5) “ Knowing the role of each team member is very good, knowing the limits when we ask for help… I'm talking about physicians asking for help, especially sometimes it's an ego issue.” (Medicine faculty 1) “Know other duties and responsibilities of each member of the team.” (Applied medical sciences student 3) “..So the point that I'm trying to raise here is understanding the roles really helps to deliver the best care possible and not following it and not following what you're supposed to do.” (Medicine student 1) “I think the most important is communication skills. During my internship in the rounds, I am having difficulties to communicate with other team members. We didn't take it in specific in undergrad.” (Applied medical sciences student 3) “Be able to communicate, to demonstrate their communication skills, especially with team conflict.” (Nursing faculty 2) “ Good communication skills, this is the first thing we should be taught about, like we will be able to have better listening, and we can communicate and learn how to provide information and how to facilitate the decision-making and coordinate the care between each of us as a team and learn from other professions.” (Medicine student 1)
